# Advancing Oral and Maxillofacial Medicine

**DOI:** 10.31661/gmj.v13iSP1.3728

**Published:** 2024-12-08

**Authors:** Seyed Masoud Sajedi

**Affiliations:** ^1^ Department of Oral and Maxillofacial Medicine, Faculty of Dentistry, Shahed University, Tehran, Iran

## Dear Readers,

It is with great excitement that we present this special issue of The Galen Medical Journal, a collection that underscores the breadth and depth of contemporary research in oral and maxillofacial medicine. This issue features a remarkable array of articles, from cutting-edge studies on diagnostic imaging and innovative surgical techniques to investigations into biomaterials, regenerative therapies, and interdisciplinary treatment approaches.

The contributions to this issue reflect the diversity of challenges and opportunities in our field. Highlights include:

## Emerging Technologies in Diagnosis and Treatment:

Articles such as "Micro-CT evaluation of marginal and internal fit of lithium disilicate crowns" and "The role of 3D printing in customizing dental prosthetics and orthodontic appliances" showcase how technology is driving precision and personalization in dental and maxillofacial care.

## Innovations in Biomaterials and Regenerative Medicine:

Studies like "Advances in regenerative medicine for the treatment of osteonecrosis of the jaw" and "Evaluation of a novel nanocomposite’s bioactivity for use in dentistry" emphasize the potential of biomaterials to transform treatment outcomes.

## Impactful Clinical and Systematic Reviews:

Comprehensive reviews, including "Trends and patterns in mandible bone cancer research" and "Systematic review of the combined treatment of endo, ortho, and prosthetics in maxillary central fracture", provide critical insights into evolving clinical practices.

## Interdisciplinary and Personalized Care:

Interdisciplinary approaches, as seen in articles like "Temporary Anchorage Devices (TADs) in combined treatments and Neurosurgical complications following tooth extraction", highlight the importance of collaboration across specialties.

This issue also brings to light essential public health concerns and their clinical implications, as evidenced by studies on the "oral and dental health of patients with rheumatoid arthritis" and "the prevalence of supernumerary teeth in patients with cleft lip and palate".

The diversity of topics reflects our commitment to fostering knowledge across the full spectrum of oral and maxillofacial research. Whether exploring advanced diagnostics, novel therapeutic modalities, or public health impacts, the articles in this issue underscore the innovative spirit and dedication of researchers in the field.

On behalf of the editorial board, I would like to extend my gratitude to the authors and reviewers who contributed their expertise and insights. This issue stands as a testament to the collective efforts of the oral and maxillofacial medical community to advance patient care and scientific understanding.

We hope that this compilation of studies will serve as a valuable resource for clinicians, researchers, and students, inspiring future investigations and fostering interdisciplinary collaborations.

Sincerely,

